# Multimodal neuroimaging exploration of the mechanisms of sleep quality deterioration after SARS-CoV-2 Omicron infection

**DOI:** 10.1186/s12916-024-03487-9

**Published:** 2024-06-26

**Authors:** Yanyao Du, Cong Li, Wei Zhao, Jinyue Li, Linlin Zhao, Huili Guo, Yingjia Jiang, Weiyin Vivian Liu, Song Zeng, Huiting Zhang, Hu Guo, Xuan Ouyang, Jun Liu

**Affiliations:** 1 Department of Radiology, Second Xiangya Hospital of Central South University, Changsha, Hunan Province 410011 China; 2 Clinical Research Center for Medical Imaging in Hunan Province, Changsha, Hunan 410011 China; 3 Department of Radiology Quality Control Center, Changsha, Hunan 410011 China; 4 Department of Psychiatry, National Clinical Research Center for Mental Disorders, and National Center for Mental Disorders, The Second Xiangya Hospital of Central South University, Changsha, Hunan Province 410011 China; 5 MR Research, GE Healthcare, Beijing, 100176 China; 6 MR Product, GE Healthcare, Guangzhou, 510000 China; 7 MR Research Collaboration, Siemens Healthineers, Wuhan, 430000 China; 8 MR Application, Siemens Healthineers, Guangzhou, 510000 China

**Keywords:** Sleep quality deterioration, Post-Omicron infection, Gray matter thickness, Cerebral edema, Perivascular space

## Abstract

**Background:**

To evaluate the neurological alterations induced by Omicron infection, to compare brain changes in chronic insomnia with those in exacerbated chronic insomnia in Omicron patients, and to examine individuals without insomnia alongside those with new-onset insomnia.

**Methods:**

In this study, a total of 135 participants were recruited between January 11 and May 4, 2023, including 26 patients with chronic insomnia without exacerbation, 24 patients with chronic insomnia with exacerbation, 40 patients with no sleep disorder, and 30 patients with new-onset insomnia after infection with Omicron (a total of 120 participants with different sleep statuses after infection), as well as 15 healthy controls who were never infected with Omicron. Neuropsychiatric data, clinical symptoms, and multimodal magnetic resonance imaging data were collected. The gray matter thickness and T1, T2, proton density, and perivascular space values were analyzed. Associations between changes in multimodal magnetic resonance imaging findings and neuropsychiatric data were evaluated with correlation analyses.

**Results:**

Compared with healthy controls, gray matter thickness changes were similar in the patients who have and do not have a history of chronic insomnia groups after infection, including an increase in cortical thickness near the parietal lobe and a reduction in cortical thickness in the frontal, occipital, and medial brain regions. Analyses showed a reduced gray matter thickness in patients with chronic insomnia compared with those with an aggravation of chronic insomnia post-Omicron infection, and a reduction was found in the right medial orbitofrontal region (mean [SD], 2.38 [0.17] vs. 2.67 [0.29] mm; *P* < 0.001). In the subgroups of Omicron patients experiencing sleep deterioration, patients with a history of chronic insomnia whose insomnia symptoms worsened after infection displayed heightened medial orbitofrontal cortical thickness and increased proton density values in various brain regions. Conversely, patients with good sleep quality who experienced a new onset of insomnia after infection exhibited reduced cortical thickness in pericalcarine regions and decreased proton density values. In new-onset insomnia patients post-Omicron infection, the thickness in the right pericalcarine was negatively correlated with the Self-rating Anxiety Scale (*r* =  − 0.538, *P* = 0.002, *P*_FDR_ = 0.004) and Self-rating Depression Scale (*r* =  − 0.406, *P* = 0.026, *P*_FDR_ = 0.026) scores.

**Conclusions:**

These findings help us understand the pathophysiological mechanisms involved when Omicron invades the nervous system and induces various forms of insomnia after infection. In the future, we will continue to pay attention to the dynamic changes in the brain related to insomnia caused by Omicron infection.

**Supplementary Information:**

The online version contains supplementary material available at 10.1186/s12916-024-03487-9.

## Background

Coronavirus disease 2019 (COVID-19), resulting from severe acute respiratory syndrome coronavirus 2 (SARS-CoV-2) infection, has led to unprecedented global morbidity and mortality. Post-viral infection neurological damage has emerged as a significant concern, giving rise to symptoms such as brain fog, insomnia, and motor retardation [1]. Previous studies have documented structural and functional brain alterations following Delta variant infection [2–5], providing valuable insights into the infection mechanisms and sequela causation. Nevertheless, a gap exists in the realm of imaging evidence, particularly concerning whether the mechanism of nervous system invasion by the currently prevalent Omicron strain parallels that of the Delta strain and whether the targeted brain regions align with those affected by the Delta variant.

Insomnia, as a symptom of neurological invasion, is a prevalent and persistent issue following Delta variant infection [6], yet our comprehension of the connection between Omicron strains and insomnia remains limited. Notably, the prevalence of insomnia is higher in Omicron-infected patients than in those infected during the dominant phase of the Delta variant or previous variants [7], and various scenarios, including the exacerbation of preexisting insomnia and the emergence of new-onset insomnia, have been documented [6]. While an imaging study has explored the link between neurologic MRI and sleep disorders post-COVID-19 [2], the association between insomnia disorders and neurological structures following Omicron variant infection, as well as the mechanisms underlying the deterioration in sleep quality and the related neurological structural changes across different populations, remains unclear.

Multimodal magnetic resonance imaging (MRI) offers a broader range of complementary physiological and biochemical insights into the brain than single-modality MRI. Nonetheless, there is a deficiency of multimodal imaging data for Omicron-infected individuals, along with a lack of comprehensive neuropsychological assessment scales. Therefore, we employed three sequences, namely, three-dimensional magnetization-prepared rapid acquisition gradient echo (3D-MPRAGE), magnetic resonance image compilation (MAGiC) [8], and diffusion tensor imaging analysis along the perivascular space (DTI-ALPS) [9], to capture structural and functional brain changes. Our overall objective was to investigate the associations of 3D-MRPAGE, MAGiC, and DTI-ALPS findings with neuropsychological factors, encompassing measurements of cortical thickness, relaxometry information, and lymphatic system activity within the brain [10].

This study had three primary objectives: First, we evaluated the changes in cortical thickness by comparing four groups of patients with different sleep statuses after Omicron infection to uninfected healthy controls, respectively. This aimed to assess the alterations in gray matter microstructure of the nervous system induced by Omicron infection. Specifically, we compared chronic insomnia with exacerbated chronic insomnia in Omicron patients and examined individuals without insomnia alongside those with new-onset insomnia. Third, we aimed to examine the neuropsychological relevance of the aforementioned changes in Omicron-infected individuals experiencing a deterioration in sleep quality. We prospectively collected imaging data, alongside pertinent clinical information and neuropsychological data, from both healthy subjects and Omicron-infected individuals with varying sleep quality. The differences in the brain were investigated in an MRI data-driven manner.

## Methods

### Participants and study design

We conducted a prospective cross-sectional study on 120 participants with different sleep statuses after infection with Omicron. All participants were consecutively recruited through the insomnia clinic in the Department of Psychiatry and WeChat recruitment advertisements between February 6 and May 4, 2023.

**Fig. 1:**
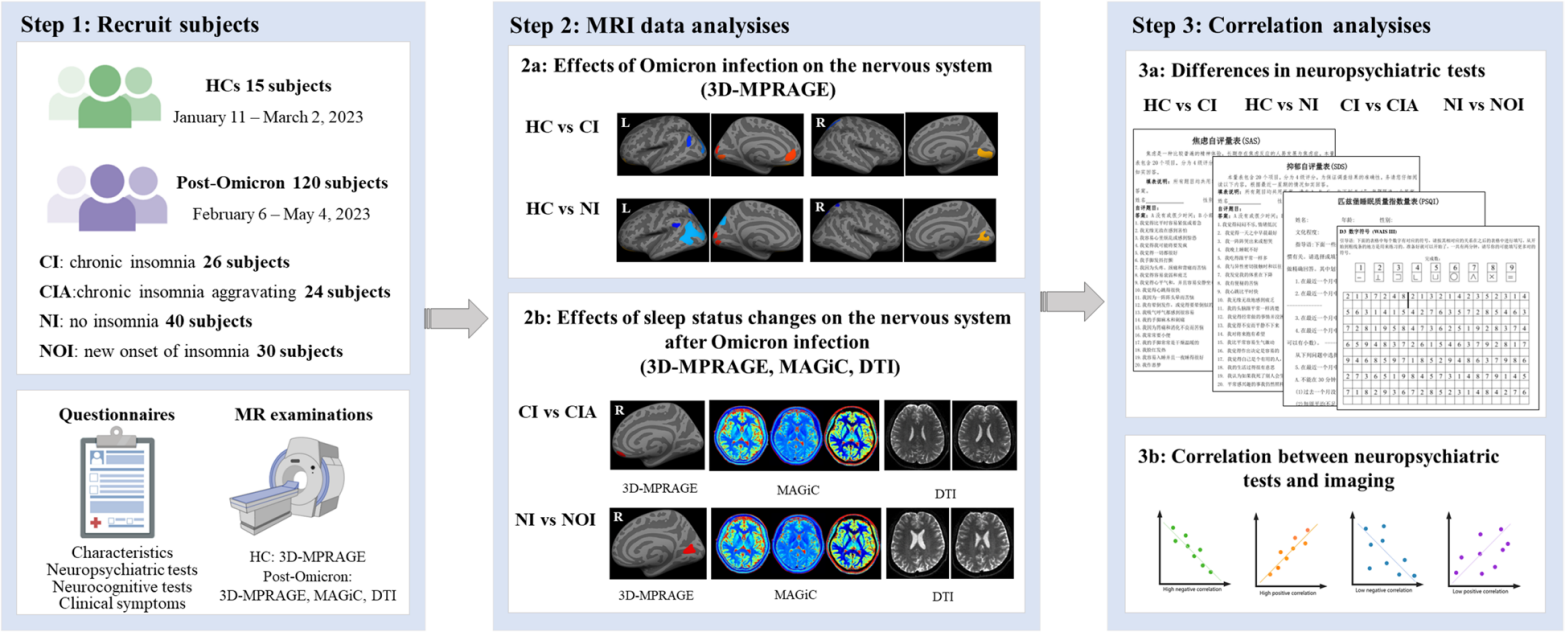
Flowchart of the study. 3D-MPRAGE, three-dimensional magnetization-prepared rapid acquisition gradient echo; MAGiC, magnetic resonance image compilation; DTI, diffusion tensor imaging

All subjects infected with Omicron were diagnosed twice by three experienced psychiatrists according to the International Classification of Sleep Disorders (ICSD-3) to determine whether they suffered from insomnia disorders before and after infection. For patients diagnosed with insomnia disorder before infection, those whose sleep disturbances and daytime symptoms persisted for more than 3 months were diagnosed with chronic insomnia. Subsequently, chronic insomnia patients were divided into groups based on subjective perceptions of changes in sleep status after Omicron infection, namely the exacerbated chronic insomnia group and the non-exacerbated chronic insomnia group. Patients who did not exhibit symptoms of insomnia before infection but developed symptoms consistent with the ICSD-3 diagnostic criteria after infection, which did not persist for more than 3 months, were diagnosed with acute insomnia. Therefore, the 120 participants infected with Omicron were divided into four groups: (a) patients with a history of chronic insomnia whose sleep status did not change after infection (CI group), (b) patients with a history of chronic insomnia whose insomnia symptoms worsened after infection (CIA group), (c) individuals with good sleep quality who had no change in sleep status after infection (NI group), and (d) individuals with good sleep quality who experienced a new onset of insomnia after infection (NOI group). Fifteen healthy controls (HCs) were also recruited through WeChat recruitment advertisements from January 11 to March 2, 2023, and they underwent regular SARS-CoV-2 nucleic acid testing to ensure they were not infected with SARS-CoV-2. The inclusion criteria for the different sleep status groups were as follows: (a) nucleic acid test result was positive; (b) the sleep state of chronic insomnia patients and individuals with good sleep quality changed or remained unchanged after infection; and (c) did not infect with Delta variant and infected with Omicron variant for the first time. The exclusion criteria for all participants were as follows: (a) an MRI contraindication; (b) a history of structural brain abnormalities, such as epilepsy, traumatic brain injury and psychiatric illness; and (c) a history of tumors and endocrine diseases. This study was approved by the Ethics Committee of the Second Xiangya Hospital of Central South University, Hunan, China (approval number: 2020S004), and all participants signed informed written consent. The experimental design is shown in Fig. 1. Comparisons were made as follows: (a) brain gray matter thickness changes were compared between the HC group and the CI group, the HC group and the CIA group, the HC group and the NI group, and the HC group and the NOI group; (b) brain gray matter thickness, the index of MAGiC, and the values of ALPS changes were compared between the CI and CIA groups and the NI and NOI groups.

### Clinical and neuropsychological tests

Demographic characteristics and neuropsychological test results were collected from 135 individuals following MRI scanning, including the Pittsburgh Sleep Quality Index (PSQI), Self-rating Anxiety Scale (SAS) score, and Self-rating Depression Scale (SDS) score. The cognitive tests administered included the Logical Memory (LM) test, the Digit Symbol Substitution Test (DSST), the Knowledge subscale of the Wechsler Intelligence scale, the Forward and Backward Digit Span (FDS and BDS, respectively) tests, and the Word Fluency Test (WFT), which was assessed in terms of logical memory, processing speed, sustained attention, and working memory. The inclusion of cognitive tests aimed to control for gray matter changes in the nervous system caused by differences in cognitive function among groups. For the one hundred and twenty post-Omicron infection patients, information on multisystem clinical symptoms, the PSQI, and the Insomnia Severity Index (ISI) were collected.

### MRI acquisition and MR data preprocessing

Patients were scanned on a 3.0-T MR scanner (Signa Premier, GE Healthcare, Waukesha, WI) using a 48-channel head coil. The MRI scanning sequences included T1-weighted imaging, T2-weighted imaging, fluid-attenuated inversion recovery imaging, 3D-MPRAGE, MAGiC, and DTI. 3D-MPRAGE of HCs was acquired using a 3.0-T MRI scanner (Siemens Skyra, Siemens Healthcare, Erlangen, Germany) with 32-channel head coils. All participants wore earplugs and were placed supine with a foam pad between the head and the coil to minimize motion artifacts. The scanning parameters are shown in Additional file 1.

### 3D-MPRAGE

The gray matter thicknesses were calculated by freesurfer 6.0 (http://surfer.nmr.mgh.harvard.edu). The preprocessing pipeline encompassed a sequence of essential procedures, commencing with the assessment of image quality and followed by motion correction; the removal of nonbrain tissues including the neck, scalp, and skull; subsequent intensity normalization; Talairach registration; precise segmentation of subcortical white matter; and meticulous demarcation of gray and white matter boundaries. Further refinement was achieved through topology correction and surface deformation processes. Ultimately, each individual’s cerebral structure was co-registered with a standardized spherical atlas. Additionally, manual interventions were carried out to rectify any inaccuracies in cortical segmentation within the image dataset.

### MAGiC

The whole-brain T1, T2, and proton density (PD) values of each patient were automatically extracted based on the anatomical labeling gray matter template and the Johns Hopkins University White Matter Template using an in-house code embedded in MATLAB 2013b (V8.2.0.701) after MAGiC data were affined to 3D T1-weighted imaging using affine parameters of 3D T1-weighted images to rigidly register, segment, and normalize into a Montreal Neuroscience Institute space. Detailed brain region information is provided in Additional file 2: Table S1 [11].

### DTI-ALPS

The DTI data were processed using FSL V. 6.0.3 (https://fsl.fmrib.ox.ac.uk/fsl). In this process, the brain mask was initially estimated, and the raw images were subsequently cropped. Eddy-current artifacts were then corrected for the computation of diffusivity values along the *x*-axis, *y*-axis, and *z*-axis directions within each image. Based on the Johns Hopkins University atlas, three regions of interest (ROIs) were positioned within the projected fiber area along the *z*-axis, the associated fiber area along the *y*-axis, and the subcortical fiber area along the *x*-axis. This procedure facilitated the computation of diffusion metrics along the *x*-axis, *y*-axis, and *z*-axis for both the left and right hemispheres. Finally, the ALPS indexes were calculated for each case: ALPS index = (mean (*x*-axis diffusivity in the projection area, *x*-axis diffusivity in the association area))/(mean (*y*-axis diffusivity in the projection area, *z*-axis diffusivity in the association area)). The ALPS indexes for the left sides and right sides of the projection areas and association areas, as well as the average bilateral ALPS indexes, were calculated [9, 12].

### Statistical analysis

Clinical information, scale scores, and T1, T2, PD, and ALPS index values were evaluated by using IBM SPSS Statistics 24.0. Quantitative data are presented as either the mean ± standard deviation or median and quartiles, while categorical data are presented as frequencies and percentages. Two-sample Wilcoxon-Mann–Whitney *U* tests, two-sample *t* tests, and chi-square tests were used in the statistical comparisons. For the 3D-MPRAGE data, multiple comparisons were corrected for using Monte Carlo simulation cluster analysis. For MAGiC and ALPS data, we applied false discovery rate (FDR) correction for multiple comparisons. Statistical significance was denoted by* P* values less than 0.05.

### Post hoc ROIs and correlation analysis

The gray matter thicknesses were extracted from each subject and displayed in a violin image. Pearson or Spearman correlation analyses were applied as appropriate based on the normal distribution of the data. Linear regression analysis was used to calculate the correlation between continuous variables and binary data. For all correlation analysis results, we also conducted FDR correction for multiple comparisons.

## Results

### Participant characteristics and clinical symptoms

The summary of participant characteristics is presented in Table 1 and Additional file 2: Table S2. There was no statistically significant difference in age, sex, education level, or neurocognitive test scores between the HC and CI groups, HC and NI groups, CI and CIA groups, and NI and NOI groups. Compared with HCs, CI patients demonstrated significantly increased PSQI (mean [SD], 5.07 [2.19] vs. 12.00 [3.25]; *P* < 0.001), SAS (mean [SD], 31.27 [7.54] vs. 39.50 [8.62]; *P* = 0.004), and SDS (mean [SD], 32.71 [7.93] vs. median, 39.00 [IQR, 33.25–50.00]; *P* = 0.005) scores, but there were no statistically significant differences in neuropsychiatric test scores between HCs and NI patients. CIA patients had significantly increased ISI (mean [SD], 10.62 [7.78] vs. 14.67 [5.49]; *P* = 0.040), SAS (mean [SD], 39.50 [8.62] vs. 46.29 [10.88]; *P* = 0.018), and SDS (median, 39.00 [IQR, 33.25–50.00] vs. mean [SD], 48.67 [10.83]; *P* = 0.010) scores compared with CI patients, but there was no significant difference in PSQI scores between the two groups. PSQI (mean [SD], 4.03 [1.98] vs. 12.97 [2.31]; *P* < 0.001), ISI (mean [SD], 2.40 [2.31] vs. 14.50 [5.53]; *P* < 0.001), SAS (mean [SD], 32.98 [7.72] vs. 47.17 [10.49]; *P* < 0.001), and SDS (median, 31.00 [IQR, 26.00–36.00] vs. mean [SD], 49.20 [11.91]; *P* < 0.001) scores were significantly different between the NI and NOI groups.

**Table 1:** Summary of participant characteristics

	**HCs (*N* = 15)**	**CI (*N* = 26)**	**CIA (*N* = 24)**	**NI (*N* = 40)**	**NOI (*N* = 30)**	**t/χ**^**2**^**/Z**	***P***_***1***_	**t/χ**^**2**^**/Z**	***P***_***2***_	**t/χ**^**2**^**/Z**	***P***_***3***_	**t/χ**^**2**^**/Z**	***P***_***4***_
Age (years)	41.87 ± 13.90^a^	50.00 (34.00, 57.00)^b^	40.04 ± 11.75	35.75 ± 12.77	39.80 ± 10.45	− 0.935	0.355	1.545	0.128	− 1.848	0.065	− 1.458	0.149
Gender (male/female)^c^	8/7	20/6	20/4	28/12	23/7	1.476	0.224	1.340	0.247	0.045	0.832	0.385	0.535
Education (years)	14.93 ± 2.05	13.32 ± 4.56	16.00 (12.00, 19.00)	16.00 (12.00, 16.00)	15.23 ± 3.01	1.529	0.135	− 0.365	0.715	− 1.157	0.247	− 0.527	0.598
BMI (kg/m^2^)	23.46 ± 3.30	23.02 ± 2.74	22.05 ± 2.70	20.92 ± 6.10	22.66 ± 4.16	0.456	0.651	1.523	0.134	1.256	0.215	− 1.346	0.183
Nicotine use (yes/no)	6/9	4/22	0/24	4/36	3/27	2.762	0.096	4.737	0.030^*^	1.335	0.248	0.000	1.000
Alcohol use (yes/no)	4/11	4/22	3/21	5/35	3/27	0.220	0.639	0.732	0.392	0.000	1.000	0.000	1.000
Handedness	15R	26R	24R	40R	30R								
Time interval between Omicron infection and MR examination (days)	-	87.70 ± 16.29	81.00 ± 23.42	93.08 ± 20.57	78.83 ± 15.75	-	-	-	-	1.126	0.266	− 3.122	0.003^*^
Years of insomnia before infection (years)	-	4.25 (2.25, 8.00)	3.00 (2.00, 8.75)	-	-	-	-	-	-	− 0.751	0.452	-	**-**
**Vaccination Status**													
Unvaccinated	0	1	1	1	0	3.301	0.347	0.720	0.868	3.537	0.316	1.616	0.655
Single- or double-vaccinated	3	2	6	8	5								
Booster vaccinated	12	23	17	31	25								
**Neuropsychiatric tests**													
PSQI	5.07 ± 2.19	12.00 ± 3.25	13.12 ± 3.01	4.03 ± 1.98	12.97 ± 2.31	− 7.341	< 0.001^*^	1.689	0.097	− 1.267	0.211	− 17.397	< 0.001^*^
ISI	-	10.62 ± 7.78	14.67 ± 5.49	2.40 ± 2.31	14.50 ± 5.53	-	-	-	-	− 2.110	0.040^*^	− 11.279	< 0.001^*^
SAS	31.27 ± 7.54	39.50 ± 8.62	46.29 ± 10.88	32.98 ± 7.72	47.17 ± 10.49	− 3.080	0.004^*^	− 0.772	0.444	− 2.456	0.018^*^	− 6.364	< 0.001^*^
SDS	32.71 ± 7.93	39.00 (33.25, 50.00)	48.67 ± 10.83	31.00 (26.00, 36.00)	49.20 ± 11.91	− 2.765	0.005^*^	− 0.379	0.705	− 2.586	0.010^*^	− 5.508	< 0.001^*^
**Neurocognitive tests**													
LM_A	8.00 ± 3.59	9.54 ± 4.18	10.92 ± 4.66	9.93 ± 4.97	10.87 ± 4.40	− 1.193	0.240	− 1.585	0.122	− 1.102	0.276	− 0.823	0.413
LM_B	6.80 ± 4.36	8.65 ± 4.21	9.67 ± 4.09	9.33 ± 5.14	9.67 ± 3.90	− 1.340	0.188	− 1.686	0.098	− 0.861	0.394	− 0.316	0.753
FDS	13.00 (12.00, 14.00)	11.00 (8.75, 13.25)	13.00 (10.50, 14.00)	12.00 (11.00, 13.00)	13.00 (10.75, 14.00)	− 1.893	0.063	− 1.073	0.283	− 1.712	0.087	− 0.370	0.711
BDS	9.27 ± 5.05	6.00 (5.00, 9.25)	7.50 ± 3.11	8.55 ± 3.15	7.97 ± 3.01	− 0.873	0.382	0.631	0.530	− 0.127	0.898	0.781	0.438
DSST	69.00 (54.00, 96.00)	75.88 ± 24.44	78.54 ± 23.74	86.23 ± 24.65	86.30 ± 17.08	− 0.257	0.797	− 1.030	0.303	− 0.389	0.699	− 0.014	0.989
Knowledge subscale of Wechsler Intelligence	21.00 (19.00, 23.00)	16.88 ± 6.67	20.00 (15.25, 26.00)	19.00 (13.50, 24.00)	20.50 (17.00, 25.00)	− 1.943	0.052	− 1.109	0.268	− 1.810	0.070	− 1.213	0.225
WFT	21.47 ± 5.44	20.00 ± 5.21	22.42 ± 8.08	21.53 ± 6.36	22.33 ± 5.19	0.855	0.398	− 0.031	0.975	− 1.246	0.220	‒0.569	0.572

*P*_*1*_: HCs vs CI. *P*_*2*_: HCs vs NI. *P*_*3*_: CI vs CIA. *P*_*4*_: NI vs NOI

*HCs* Healthy controls, *CI* Patients with a history of chronic insomnia whose sleep status did not change after infection, *CIA* Patients with a history of chronic insomnia whose insomnia symptoms worsened after infection, *NI* Individuals with good sleep quality who had no change in sleep status after infection, *NOI* Individuals with good sleep quality who experienced a new onset of insomnia after infection, *N* Number of subjects, *BMI* Body mass index, *R* Right, *PSQI* Pittsburgh Sleep Quality Index, *ISI* Insomnia Severity Index, *SAS* Self-rating Anxiety Scale, *SDS* Self-rating Depression Scale, *LM* Logical memory task, *FDS* Forward digit span, *BDS* Backward digit span, *DSST* Digital symbol substitution test, *WFT* Word fluency test

^*^P values less than 0.05 indicate statistical significance

^a^The statistical expression was mean ± SD using a two-sample *t* test

^b^The statistical expression was M(P_25_, P_75_) using Wilcoxon‒Mann‒Whitney *U* test

^c^Chi-square test

As shown in Table 2, there were no significant differences in the clinical symptoms between CI and CIA participants. Compared with respiratory and digestive system symptoms, patients with a history of chronic insomnia had a higher frequency of neurological symptoms after being infected with Omicron, such as fever (CI vs. CIA, 16 participants [61.53%] vs. 16 [66.67%]), headaches (14 [53.85%] vs. 16 [66.67%]), fatigue (18 [69.23%] vs. 22 [91.67%]), myalgia (15 [57.69%] vs. 14 [58.33%]), and taste loss (15 [57.69%] vs. 14 [58.33%]). Among the respiratory and digestive system symptoms, cough (CI vs. CIA, 14 participants [53.85%] vs. 18 [75.00%]) and decreased appetite (13 [50.00%] vs. 16 [66.67%]) had the highest incidence. Compared with the NI group, the NOI group showed a significant increase in the frequency of headache (NI vs. NOI, 23 participants [57.50%] vs. 25 [83.33%]; *P* = 0.021), myalgia (22 [55.00%] vs. 24 [80.00%]; *P* = 0.029), slower reaction time (9 [22.50%] vs. 16 [53.33%]; *P* = 0.008), expectoration (17 [42.50%] vs. 24 [80.00%]; *P* = 0.002), dyspnea (4 [10.00%] vs. 10 [33.33%]; *P* = 0.016), and chest tightness (5 [12.50%] vs. 13 [43.33%]; *P* = 0.003).

**Table 2:** Clinical information between CI and CIA groups and NI and NOI groups

	**CI (N, %)**	**CIA (*N*, %)**	**χ**^**2**^	***P***^a^	**NI (*N*, %)**	**NOI (N, %)**	**χ**^**2**^	***P***^a^
Fever	16, 61.53%	16, 66.67%	0.142	0.706	33, 82.50%	26, 86.67%	0.02	0.887
Headache	14, 53.85%	16, 66.67%	0.855	0.355	23, 57.50%	25, 83.33%	5.309	0.021^*^
Fatigue	18, 69.23%	22, 91.67%	2.649	0.104	32, 80.00%	28, 93.33%	1.519	0.218
Myalgia	15, 57.69%	14, 58.33%	0.002	0.963	22, 55.00%	24, 80.00%	4.755	0.029^*^
Olfactory loss	7, 26.92%	7, 29.17%	0.031	0.86	13, 32.50%	10, 33.33%	0.005	0.941
Taste loss	15, 57.69%	14, 58.33%	0.002	0.963	21, 52.50%	17, 56.67%	0.12	0.729
Slower reaction time	10, 38.46%	11, 45.83%	0.278	0.598	9, 22.50%	16, 53.33%	7.099	0.008^*^
Motor delay	4, 15.38%	9, 37.50%	3.172	0.075	18, 20.00%	12, 40.00%	3.36	0.067
Cough	14, 53.85%	18, 75.00%	2.424	0.119	28, 70.00%	25, 83.33%	1.657	0.198
Expectoration	10, 38.46%	12, 50.00%	0.674	0.412	17, 42.50%	24, 80.00%	9.935	0.002^*^
Dyspnea	6, 23.08%	7, 29.17%	0.241	0.624	4, 10.00%	10, 33.33%	5.833	0.016^*^
Chest tightness	5, 19.23%	8, 33.33%	1.29	0.256	5, 12.50%	13, 43.33%	8.532	0.003^*^
Chest pain	1, 9.80%	3, 12.50%	0.366	0.545	3, 7.50%	5, 16.67%	0.662	0.416
Decreased appetite	13, 50.00%	16, 66.67%	1.423	0.233	24, 60.00%	22, 73.33%	1.353	0.245
Nausea	1, 9.80%	3, 12.50%	0.366	0.545	9, 22.50%	4, 13.33%	0.953	0.329

*CI* Patients with a history of chronic insomnia whose sleep status did not change after infection, *CIA* Patients with a history of chronic insomnia whose insomnia symptoms worsened after infection, *NI* Individuals with good sleep quality who had no change in sleep status after infection, *NOI* Individuals with good sleep quality who experienced a new onset of insomnia after infection, *N* Number of subjects

^*^P values less than 0.05 indicate statistical significance

^a^Chi-square test

### Multimodal brain MRI alterations

#### Effects of Omicron infection on the nervous system

**Fig. 2:**
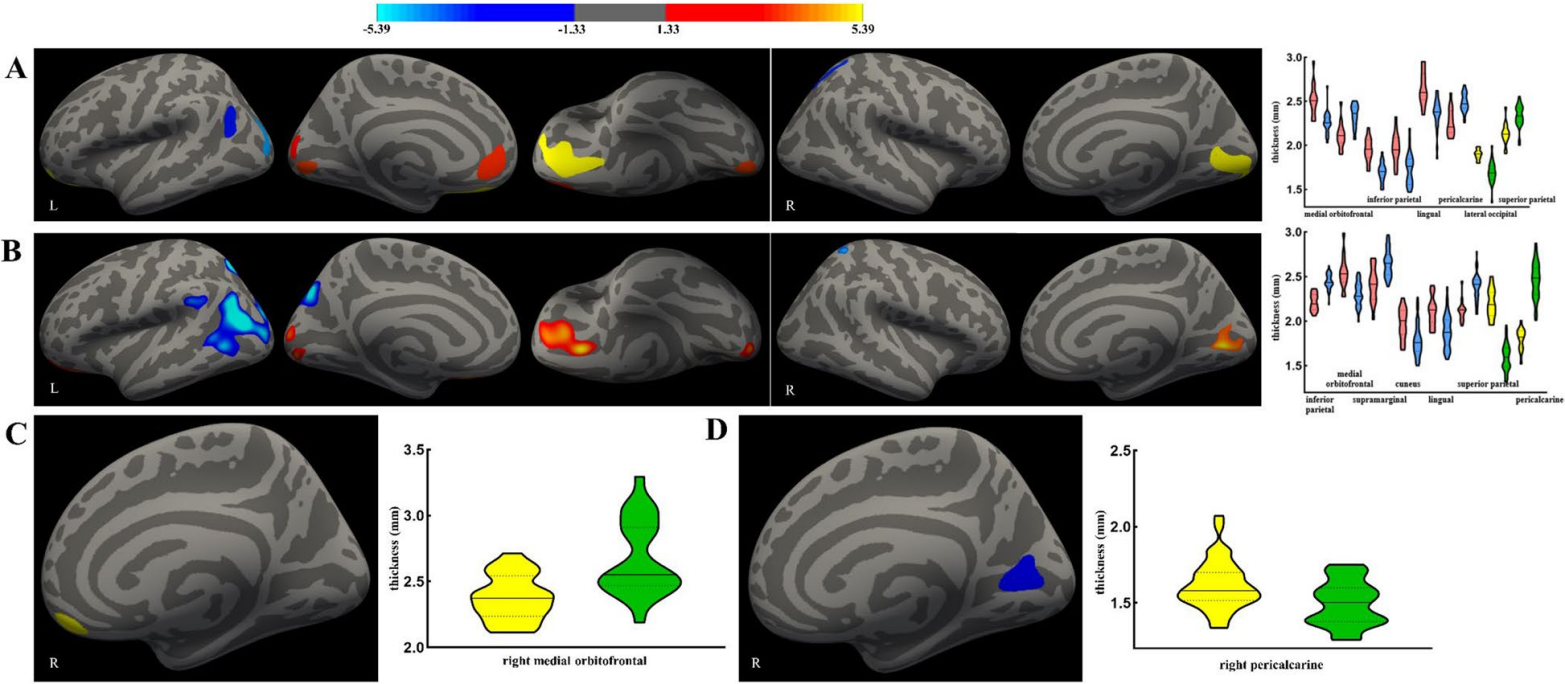
Results of gray matter thickness analysis and post hoc ROI analysis between HCs and CI groups, HCs and NI groups, CI and CIA groups, and NI and NOI groups. **A** Compared with the HCs, the gray matter thickness significantly reduced in the left medial orbitofrontal, lingual, pericalcarine, and right lateral occipital and significantly increased in the left inferior parietal and right superior parietal in the CI group. **B** Compared with the HCs, the NI group had significantly reduced gray matter thickness of the left medial orbitofrontal and lingual and increased in the left inferior parietal, supramarginal, and bilateral superior parietal. **C** Significant difference in the right medial orbitofrontal between CI and CIA groups. **D** NOI patients revealed reduced gray matter thickness in the right pericalcarine compared to NI group. In the violin image, red and blue showed the gray matter thickness difference in the left brain, and yellow and green showed the gray matter thickness difference in the right brain. ROI, region of interest; R, right; L, left; CI, patients with a history of chronic insomnia whose sleep status did not change after infection; CIA, patients with a history of chronic insomnia whose insomnia symptoms worsened after infection; NI, individuals with good sleep quality who had no change in sleep status after infection; NOI, individuals with good sleep quality who experienced a new onset of insomnia after infection

Compared with HCs, the gray matter thickness showed a significant reduction in the left medial orbitofrontal (HCs vs. CI, mean [SD], 2.52 [0.18] to 2.26 [0.12] and 2.65 [0.19] to 2.36 [0.18] mm; *P* < 0.001 for both), lingual (mean [SD], 1.96 [0.13] to 1.70 [0.11] mm; *P* < 0.001), pericalcarine (mean [SD], 1.97 [0.18] to 1.74 [0.18] mm; *P* < 0.001) and right lateral occipital (mean [SD], 1.90 [0.06] to 1.70 [0.12] mm; *P* < 0.001) regions, and a significant increase in the left inferior parietal (mean [SD], 2.11 [0.15] to 2.34 [0.13] and 2.26 [0.15] to 2.49 [0.11] mm; *P* < 0.001 for both) and right superior parietal (mean [SD], 2.13 [0.13] to 2.33 [0.13] mm; *P* < 0.001) regions in the CI group (Fig. 2A). The NI group showed a significantly reduced gray matter thickness of the left medial orbitofrontal (HCs vs. NI, mean [SD], 2.54 [0.18] to 2.30 [0.13] mm; *P* < 0.001), cuneus (mean [SD], 1.98 [0.17] to 1.81 [0.19] mm; *P* = 0.001), lingual (mean [SD], 2.11 [0.17] to 1.85 [0.20] mm; *P* < 0.001), and right pericalcarine (mean [SD], 1.80 [0.12] to 1.59 [0.14] mm; *P* < 0.001) regions and an increased gray matter thickness in the left inferior parietal (mean [SD], 2.21 [0.10] to 2.45 [0.08] mm; *P* < 0.001), supramarginal (mean [SD], 2.41 [0.20] to 2.65 [0.15] mm; *P* < 0.001), and bilateral superior parietal (L, mean [SD], 2.12 [0.12] to 2.39 [0.16] mm and R, 2.20 [0.16] to 2.43 [0.20]; *P* < 0.001 for both) regions compared with the HC group (Fig. 2B). Details and post hoc ROI analysis are shown in Table 3. There was no correlation between the gray matter thickness and the neuropsychiatric test scores (SAS and SDS scores) in the CI group. The differences between the HC and CIA groups and the HC and NOI groups are shown in Additional file 2: Table S3.

**Table 3:** Significant gray matter thickness differences between HCs and CI groups, HCs and NI groups, CI and CIA groups, and NI and NOI groups

**Index**	**Brain regions**	**Side (R/L)**	**Size**	**MNI coordinate**			**Values**		***P***
				**X**	**Y**	**Z**			
**HCs vs CI (15 vs 26)**									
Thickness (mm)	Medial orbitofrontal	L	934.09	− 11.9	25.2	− 16.9	2.52 ± 0.18	2.26 ± 0.12	< 0.001
			415.82	− 10.3	39.9	− 4.2	2.65 ± 0.19	2.36 ± 0.18	< 0.001
	Inferior parietal	L	523.47	− 28.9	− 84.2	12.2	2.11 ± 0.15	2.34 ± 0.13	< 0.001
			380.10	− 39.2	− 62.4	29.7	2.26 ± 0.15	2.49 ± 0.11	< 0.001
	Lingual	L	462.16	− 3.7	− 87.1	− 5.4	1.96 ± 0.13	1.70 ± 0.11	< 0.001
	Pericalcarine	L	332.09	− 7.0	− 93.7	8.2	1.97 ± 0.18	1.74 ± 0.18	< 0.001
	Lateral occipital	R	1542.30	22.6	− 91.7	− 7.9	1.90 ± 0.06	1.70 ± 0.12	< 0.001
	Superior parietal	R	423.87	20.2	− 59.2	55.4	2.13 ± 0.13	2.33 ± 0.13	< 0.001
**HCs vs NI (15 vs 40)**									
Thickness (mm)	Inferior parietal	L	3835.17	− 40.2	− 70.0	14.5	2.21 ± 0.10	2.45 ± 0.08	< 0.001
	Medial orbitofrontal	L	803.98	− 12.5	26.1	− 17.1	2.54 ± 0.18	2.30 ± 0.13	< 0.001
	Supramarginal	L	296.95	− 52.3	− 41.4	31.3	2.41 ± 0.20	2.65 ± 0.15	< 0.001
	Cuneus	L	283.66	− 4.9	− 92.7	9.1	1.98 ± 0.17	1.81 ± 0.19	0.001
	Lingual	L	276.64	− 6.8	− 92.7	− 10.2	2.11 ± 0.17	1.85 ± 0.20	< 0.001
	Superior parietal	L	382.95	− 16.9	− 65.1	50.2	2.12 ± 0.12	2.39 ± 0.15	< 0.001
		R	233.91	29.3	− 49.0	63.5	2.20 ± 0.16	2.43 ± 0.20	< 0.001
	Pericalcarine	R	644.63	8.9	− 72.8	5.7	1.80 ± 0.12	1.59 ± 0.14	< 0.001
**CI vs CIA (26 vs 24)**									
Thickness (mm)	Medial orbitofrontal	R	226.42	5.9	47.1	− 18.3	2.38 ± 0.17	2.67 ± 0.29	< 0.001
**NI vs NOI (40 vs 30)**									
Thickness (mm)	Pericalcarine	R	705.06	23.5	− 69.5	4.0	1.62 ± 0.16	1.50 ± 0.15	0.002

*R* Right, *L* Left, *MNI* Montreal Neurological Institute, *HCs* Healthy controls, *CI* Patients with a history of chronic insomnia whose sleep status did not change after infection, *CIA* Patients with a history of chronic insomnia whose insomnia symptoms worsened after infection, *NI* Individuals with good sleep quality who had no change in sleep status after infection, *NOI* Individuals with good sleep quality who experienced a new onset of insomnia after infection

#### Effects of sleep status changes on the nervous system after Omicron infection

**Fig. 3:**
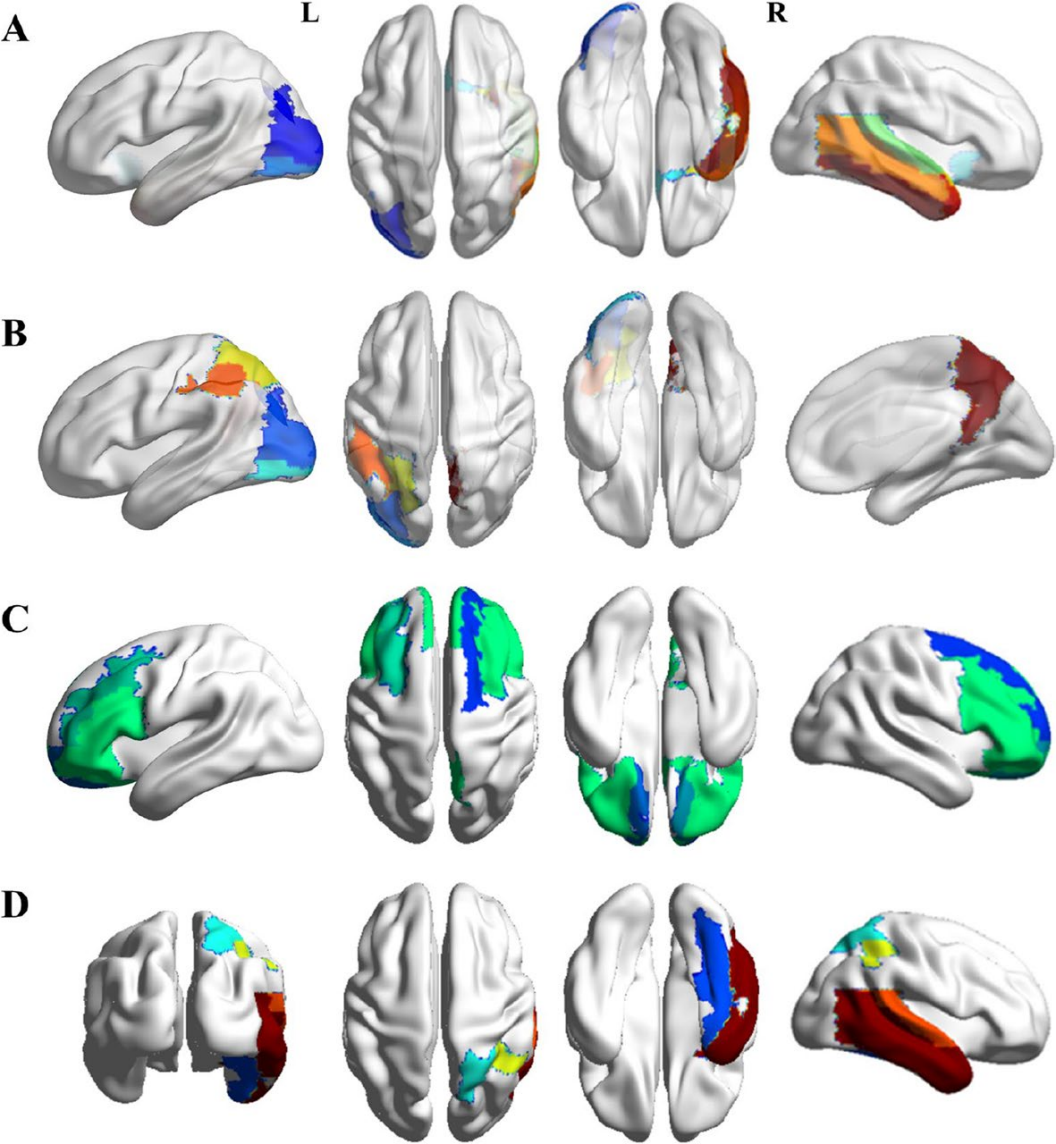
Results of MAGiC analysis between CI and CIA groups and NI and NOI groups. Compared with the CI group, the CIA group showed a significant decrease in T1 values in the left occipital and right olfactory and temporal lobes (**A**) and an increase in T2 values in the left occipital and parietal, and right precuneus lobes (**B**), and in PD values in the bilateral frontal and right occipital and precuneus lobes (**C**). **D** The NOI group had a significant decrease in PD values in the right lingual, fusiform, parietal, and temporal lobes) compared with the NI group. CI, patients with a history of chronic insomnia whose sleep status did not change after infection; CIA, patients with a history of chronic insomnia whose insomnia symptoms worsened after infection; NI, individuals with good sleep quality who had no change in sleep status after infection; NOI, individuals with good sleep quality who experienced a new onset of insomnia after infection; MAGiC, magnetic resonance image compilation; PD, proton density

3D-MPRAGE data analyses showed a lower gray matter thickness in the CI group than in the CIA group, and a reduction was found in the right medial orbitofrontal region (CI vs. CIA, mean [SD], 2.38 [0.17] to 2.67 [0.29] mm; *P* < 0.001) (Fig. 2C). Compared with the CI group, the CIA group showed a significant decrease in T1 values (left occipital and right olfactory and temporal lobes) and an increase in T2 values (left occipital and parietal and right precuneus lobes) and PD values (bilateral frontal and right occipital and precuneus lobes) (Fig. 3). ALPS-L had a significant increase in the CI (L vs R, mean [SD], 1.55 [0.24] to 1.38 [0.19]; *P* < 0.001) and CIA (L vs R, mean [SD], 1.53 [0.27] to 1.36 [0.18]; *P* < 0.001) patients compared with ALPS-R. Details and post hoc ROI analysis results are shown in Tables 3 and 4. There was no correlation between the gray matter thickness and neuropsychiatric test scores (SAS and SDS scores) in the CIA patients.

**Table 4:** Significant MAGiC and DTI index differences between CI and CIA groups and NI and NOI groups

Index	Brain regions	Side (R/L)	Values		t	*P*
**CI vs CIA (26 vs 24)**						
T1^*^	Occipital	L	1196.50 ± 78.43	1152.64 ± 38.64	2.537	0.016^*^
	Olfactory	R	1242.96 ± 71.79	1206.46 ± 45.21	2.168	0.036^*^
	Temporal	R	1416.05 ± 51.95	1381.10 ± 42.65	0.146	0.013^*^
T2^*^	Occipital	L	94.90 ± 4.11	100.28 ± 10.88	− 2.345	0.025^*^
	Parietal	L	111.33 ± 9.86	118.13 ± 12.36	− 2.137	0.038^*^
	Precuneus	R	95.11 ± 4.75	98.35 ± 6.47	− 2.024	0.049^*^
PD^*^	Frontal	L	79.03 ± 2.03	80.59 ± 2.46	− 2.458	0.018^*^
		R	85.32 ± 3.59	87.81 ± 2.44	− 2.838	0.007^*^
	Olfactory	R	80.72 ± 2.15	81.93 ± 1.37	− 2.347	0.023^*^
	Precuneus	R	76.37 ± 1.28	77.21 ± 1.11	− 2.458	0.018^*^
** ALPS-L**	-	L	1.55 ± 0.24	1.53 ± 0.27	0.308	0.759
** ALPS-R**	-	R	1.38 ± 0.19	1.36 ± 0.18	0.414	0.681
** ALPS L vs R (CI)**	-	-	1.55 ± 0.24 (L)	1.38 ± 0.19 (R)	5.839	< 0.001^*^
** ALPS L vs R (CIA)**			1.53 ± 0.27 (L)	1.36 ± 0.18 (R)	5.164	< 0.001^*^
** ALPS-mean**	-	-	1.46 ± 0.20	1.44 ± 0.22	0.351	0.727
**NI vs NOI (40 vs 30)**						
PD^*^	Lingual	R	76.81 ± 0.78	76.54 ± 1.05	2.881	0.005^*^
	Fusiform	R	80.72 ± 1.21	79.90 ± 1.28	2.738	0.008^*^
	Parietal	R	81.68 ± 1.22	80.55 ± 1.30	3.724	< 0.001^*^
	Temporal	R	82.89 ± 2.05	81.84 ± 1.63	2.326	0.023^*^
** ALPS-L**	-	L	1.49 ± 0.23	1.49 ± 0.23	0.064	0.949
** ALPS-R**	-	R	1.36 ± 0.16	1.37 ± 0.17	− 0.442	0.660
** ALPS L vs R (NI)**	-	-	1.49 ± 0.23 (L)	1.36 ± 0.16 (R)	5.832	< 0.001^*^
** ALPS L vs R (NOI)**	-	-	1.49 ± 0.23 (L)	1.37 ± 0.17 (R)	3.042	0.005^*^
** ALPS-mean**	-	-	1.42 ± 0.18	1.43 ± 0.18	− 0.118	0.907

*MAGiC* Magnetic resonance image compilation, *DTI* Diffusion tensor imaging, *R* Right, *L* Left, *MNI* Montreal Neurological Institute, *CI* Patients with a history of chronic insomnia whose sleep status did not change after infection, *CIA* Patients with a history of chronic insomnia whose insomnia symptoms worsened after infection, *NI* Individuals with good sleep quality who had no change in sleep status after infection, *NOI* Individuals with good sleep quality who experienced a new onset of insomnia after infection, *ALPS* Analysis along the perivascular space

^*^P values less than 0.05 indicate statistical significance

An analysis of NOI patients revealed reduced gray matter thickness in the right pericalcarine region (NI vs. NOI, mean [SD], 1.62 [0.16] to 1.50 [0.15] mm; *P* =0.002) compared to NI patients (Fig. 2D). The NOI group had a significant decrease in PD values (right lingual, fusiform, parietal, and temporal lobes) compared with the NI group (Fig. 3), but there was no significant difference in T1 and T2 values. The results of the MAGiC among groups did not pass FDR correction.

**Fig. 4:**
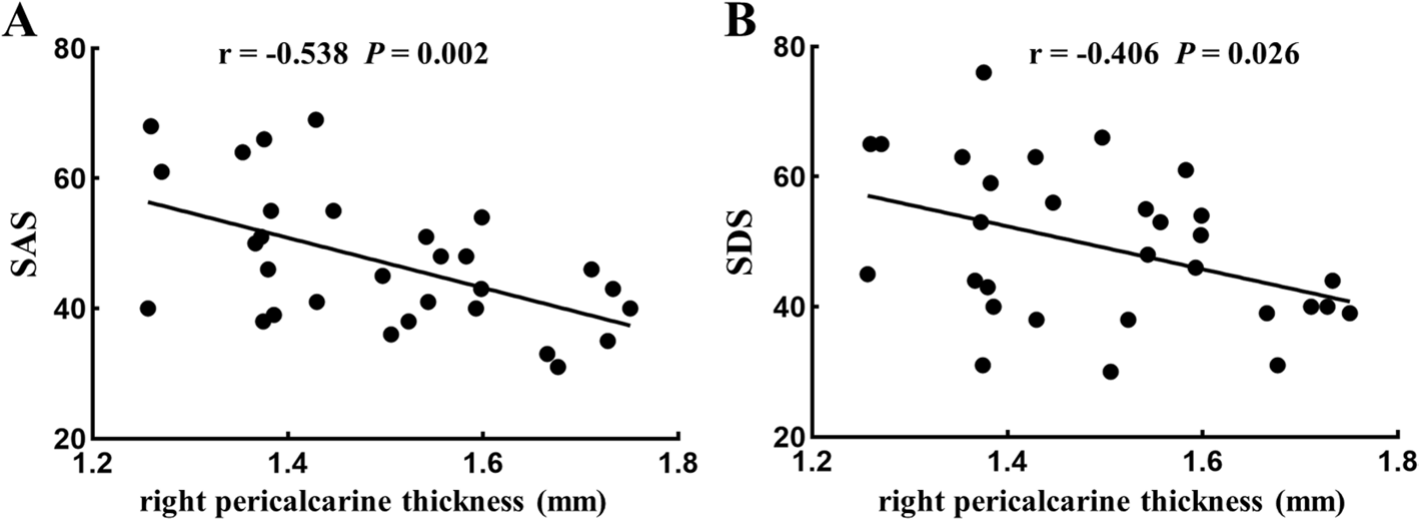
Correlation analysis results. In the NOI group, the thickness in the right pericalcarine was negatively correlated with SAS and SDS. SAS, self-rating anxiety scale; SDS, self-rating depression scale

There were no significant differences in ALPS-L, ALPS-R, and ALPS-mean scores between the CI and CIA groups or the NI and NOI groups. But compared with ALPS-R, ALPS-L showed a significant increase in the NI (L vs R, mean [SD], 1.49 [0.23] to 1.36 [0.16]; *P* < 0.001) and NOI groups (L vs R, mean [SD], 1.49 [0.23] to 1.37 [0.17]; *P* =0.005). Details and post hoc ROI analysis results are shown in Tables 3 and 4. In NOI, the thickness in the right pericalcarine was negatively correlated with SAS (Fig. 4A, *r* =  − 0.538, *P* = 0.002, *P*_FDR_ = 0.004) and SDS (Fig. 4B, *r* =  − 0.406, *P* = 0.026, *P*_FDR_ = 0.026) scores. No significant correlation was found between differential clinical symptoms in the NI and NOI groups and differential cortical thickness in brain regions.

## Discussion

This study employed a multimodal MRI approach to investigate changes in gray matter thickness in individuals with and without Omicron infection and with and without a decline in sleep quality. Comparative analysis with an HC group revealed that both CI and NI individuals exhibited similar patterns of gray matter thickness alterations. These patterns included an increase in cortical thickness near the parietal lobe and a reduction in cortical thickness in the frontal, occipital, and medial brain regions. In the subgroups of Omicron patients experiencing sleep deterioration, CIA patients displayed heightened medial orbitofrontal cortical thickness and increased PD values in various brain regions. Conversely, NOI patients exhibited reduced cortical thickness in pericalcarine regions and decreased PD values, with cortical thickness demonstrating a negative correlation with anxiety and depression scores. The findings from DTI-ALPS indicated a lateralized distribution, with all patients displaying decreased DTI-ALPS values in their right hemisphere compared to the left hemisphere.

### Neuropsychiatric symptoms and clinical sequelae

Patients infected with the Omicron variant exhibited varying degrees of exacerbation in anxiety when compared to a control group of healthy subjects. These findings are consistent with prior research [13]. Irrespective of the patient type, those experiencing a decline in sleep quality demonstrated significant differences in anxiety and depression levels compared to their pre-worsening condition. Notably, the disparity was most pronounced when contrasting the NOI group with the NI group, whereas no such difference was discerned between NI patients and HCs. Patients grappling with insomnia displayed higher levels of anxiety and depression than those without this condition [14]. These results suggest that anxiety and depressive symptoms are likely linked to Omicron-induced insomnia rather than the infection itself. Furthermore, chronic insomnia represents a substantial risk factor for the onset of anxiety and depression [15]. It is plausible that individuals in the CIA group already had elevated levels of anxiety and depression prior to the exacerbation of their insomnia, which may have contributed to the observed lesser differences. Among the symptoms assessed, insomnia patients frequently experienced fatigue, fever, cough, headache, myalgia, and decreased appetite, listed in order of frequency. The NOI group exhibited more pronounced symptoms in the nervous, respiratory, and musculoskeletal systems than the NI group. These intensified symptoms likely hindered patients from achieving restful sleep and consequently contributed to a decline in overall sleep quality.

### Effects of infection on patients with CI and NI

Our findings indicate that structural brain changes resulting from Omicron infection are characterized by similarities in individuals in the CI and NI groups. Specifically, we observed multiple regions displaying a reduction in cortical thickness, primarily concentrated in the frontal, temporal, and occipital lobes. These regions included the middle orbitofrontal lobe, lingual gyrus, pericalcarine, and cuneus. Prior investigations have reported a decline in cerebral blood flow in the frontal lobe three months after recovery in patients with severe COVID-19 [16]. The reduction in frontal lobe volume was correlated with elevated inflammatory markers during hospitalization [16], possibly indicating mechanisms associated with the immune response or inflammatory cascades. Similarly, a brain MRI study conducted on UK Biobank COVID-19 patients noted reduced gray matter thickness in the orbitofrontal cortex, even in those with milder cases [17]. SARS-CoV-2 can access the brain through various routes, including the olfactory bulb, cerebrospinal fluid, and compromised blood–brain barriers [18]. Although direct infection of the nervous system by SARS-CoV-2 is rare, relevant autopsies have found the presence of the virus in nerve and capillary endothelial cells in the frontal lobe tissues of SARS-CoV-2-infected patients [19]. The olfactory tract extends from the olfactory bulb along the base of the frontal cortex [20], and the target of SARS-CoV-2 virus, angiotensin-converting enzyme 2, is commonly expressed in vessels of various calibers in the frontal cortex [21]. Extensive connectivity exists between the occipital lobe and the olfactory bulb [22], and the amygdala, located in the medial part of the temporal lobe, receives inputs from the olfactory bulb and the associated cortex [23]. During viral brain infections, microglia and infiltrating macrophages secrete various inflammatory factors aimed at viral clearance. However, an excessive release of inflammatory factors can induce neuronal apoptosis, leading to a severe central nervous system inflammatory response and neuronal damage within the cortex [24], where neurons converge. The Omicron viral particles are likely propagated through the olfactory bulb and its connections with the frontal lobe, similar to previous strains, thus inciting neuroinflammation and neuronal destruction in these regions. These mechanisms may underlie the observed reduction in cortical thickness.

Furthermore, post-Omicron infection, patients exhibited an increase in cortical thickness in regions proximate to the parietal lobe, including the superior parietal lobe, inferior parietal lobe, and supramarginal gyrus. Notably, previous neuroimaging studies focusing on COVID-19-related neuroimaging have reported extensive white matter edema in the parietal lobe [25] and perfusion deficits [26], but no prior research has documented alterations in the parietal cortex. COVID-19 can induce neurological damage through a variety of mechanisms. Immune responses to SARS-CoV-2 can trigger neuroinflammation, leading to an elevation in cytokines, chemokines, and immune cell transport within the brain. This cascade of events induces a reactive state in microglia and other immune cells in the brain and its borders [27]. The proliferation of associated immune cells in the cortex may represent one of the mechanisms contributing to the observed increase in cortical thickness. In-depth exploration of the associated mechanisms is imperative to uncover the underlying intricacies.

Given the prior observation of reduced cortical thickness in the orbitofrontal cortex among individuals with a history of insomnia [28], there exists a potential overlap in the cortical areas affected by both insomnia and the Omicron variant. The changes in cortical thickness observed in the CI group are likely a result of the combined impact of insomnia and Omicron infection. Further investigation is required to elucidate the precise mechanisms and effects.

### Mechanisms of insomnia exacerbation in CI

In a comparison between patients with chronic insomnia exacerbation and chronic insomnia, we observed an increase in the thickness of the right medial orbitofrontal cortex in patients with CIA. However, this increase in cortical thickness was not significantly correlated with neuropsychological test scores. In further analyses using MAGiC, we identified elevated values for T2 and PD in several brain regions. Notably, PD values can offer insights into tissue water content and reflect structural damage within the brain [29]. The increased PD values observed in the MAGiC analyses may indicate higher water content in the frontal lobe. The entry of SARS-CoV-2 Omicron viral particles may have induced edema in the medial orbitofrontal cortex through the mechanisms previously discussed, potentially resulting in functional impairment in this region. This, in turn, may mediate the exacerbation of sleep disorders in patients with insomnia. Notably, prior studies have revealed a positive correlation between the thickness of the right orbitofrontal cortex and the severity of primary insomnia in patients [30]. In line with this, our findings demonstrated that individuals with CIA, experiencing exacerbated insomnia, exhibited increased thickness in the right medial orbitofrontal cortex. This finding suggests that the initially affected brain region may be more susceptible to viral impact. However, some studies have reported contrasting results, including bilateral gray matter deficits in the frontal lobes and smaller orbitofrontal cortex volumes [31] or thicknesses [28] in patients with more severe insomnia. We believe that these variations in results may be attributed to differences in subject characteristics, sample size, or variations in the mechanisms of nervous system damage. In summary, our results provide further insights into previous research, emphasizing the pivotal role of the right medial orbitofrontal cortex in the further deterioration of sleep quality induced by the Omicron variant in patients with CI.

### Mechanisms of new-onset insomnia in NI participants

The pericalcarine region of patients after Omicron infection demonstrated reduced cortical thickness and showed increased anxiety and depressive symptoms compared to HC. Further comparisons of NOI with NI, we observed reduced cortical thickness in the right pericalcarine regions and significantly higher levels of anxiety and depression in patients with NOI. Relevant studies on the pericalcarine region are relatively limited. In a comprehensive MRI study involving the brains of 6503 patients with bipolar disorder and healthy subjects, it was observed that, in adult bipolar disorder patients, an extended duration of the disease was associated with the reduced cortical thickness of the pericalcarine in the brain bilaterally [32]. In addition, studies have shown that patients with major depressive disorder have reduced gray matter volume in the right pericalcarine [33]. Similarly, our correlation analysis showed that lower right pericalcarine cortical thickness was associated with higher levels of anxiety and depression in patients with NOI. In the context of MAGiC analysis, diminished PD values were observed in several brain regions. This suggests that, unlike the situation observed in CIA, the mechanism underlying NOI may not be related to edema in key brain regions but rather linked to symptoms such as anxiety and depression. Prior research has consistently shown that mental health conditions, particularly depression and anxiety, are linked to sleep disturbances following SARS-CoV-2 infection [34–36]. The relationship between insomnia and anxiety and depression appears to be bidirectional, as insomnia can contribute to the development of anxiety and depression symptoms. Conversely, the presence of anxiety and depression can disrupt sleep patterns [37–39]. Our findings indicate that Omicron infection led to damage in the cortex of the pericalcarine area, which may be associated with negative emotions such as anxiety and depression. A combination of factors, including heightened anxiety and depression levels and a more pronounced set of clinical symptoms in patients with NOI, likely mediated the deterioration of sleep quality.

### Exploration of possible mechanisms of deteriorating sleep quality

While we did not observe significant differences in DTI-ALPS values between participants with varying sleep quality, we identified significant variations in DTI-ALPS values between the left and right hemispheres in all patients. This damage was predominantly localized on the right side, consistent with a prior study on COVID-19 brain imaging that reported a tendency for damage to be concentrated in the right cerebral hemisphere [40]. SARS-CoV-2 can induce neurological damage through neuroinflammatory mechanisms [27]. The glymphatic system functions to transport fluid to all regions of the brain, and any disruption in this system has the potential to impact the brain’s drainage capabilities [41]. A recent study on the response of the choroid plexus to local and peripheral inflammation has indicated that acute inflammation is associated with an overproduction of cerebrospinal fluid [42]. Increased cerebrospinal fluid secretion can enhance the clearance of antigens such as viruses, particularly following localized injury. Moreover, as blood flow and blood volume are typically higher in the dominant hemisphere of the right hand, the dominance of the left glymphatic system may be associated with differences in bilateral hemispheric blood volume [43]. Increased cerebral blood volume can stimulate the metabolism and activity of choroid plexus cells [44], potentially leading to heightened cerebrospinal fluid secretion. Lower DTI-ALPS values in the right hemisphere of the brain may signify a less effective clearance response following SARS-CoV-2 infection, which could explain the lateralized distribution observed in our results. Further exploration of the relevant mechanisms warrants investigation in the future. Unfortunately, DTI sequences were not collected from the HC patients in our study, which limited our ability to make comparisons.

This study has several limitations. First, due to challenges in recruiting healthy subjects who were not previously infected given the surge in BA.5/BF.7 breakthrough infections among Chinese residents between December 2022 and January 2023, we opted to include healthy subjects who had participated in a previous study as our HC group. The determination of exacerbated insomnia symptoms in patients relied on their subjective self-assessments, which may vary due to the differing sensitivities of individuals to these symptoms. The results of the MAGiC groups did not pass multiple comparison correction; however, they may reflect a trend in cortical T1, T2, and PD value changes across the groups, indicating the potential utility of the MAGiC sequence. In the future, we aim to increase the sample size to increase the statistical power, thereby allowing us to detect changes in MAGiC. Finally, and importantly, this study is cross-sectional in nature, which limits our ability to establish causal relationships. In the future, we plan to conduct longitudinal follow-up studies to provide a more comprehensive and dynamic understanding of these factors.

## Conclusions

In summary, individuals with CI and NI exhibited comparable alterations in cortical thickness relative to HCs. However, considerable disparities in cortical thickness changes and MAGiC features were evident in patients with varying sleep quality, and the negative correlation between cortical thickness and anxiety-depressive symptoms in those with NOI suggests distinct mechanisms underlying sleep quality deterioration in CI and NI patients. These findings contribute to our understanding of the pathophysiological mechanisms involved when SARS-CoV-2 invades the nervous system and leads to different forms of insomnia following infection. They also offer novel insights into the early clinical diagnosis of distinct types of sleep quality deterioration through multimodal imaging, opening avenues to target these diverse mechanisms for treatment. In the future, our research will continue to include dynamic tracking, with a specific focus on the neurological consequences associated with insomnia symptoms triggered by the Omicron variant.

### Supplementary Information

Additional file 1. 3D-MPRAGE, MAGiC and DTI scan parameters.

Additional file 2: Table S1. Detailed 46 brain region information. Table S2. Demographic characteristics of groups HCs and CIA, HCs and NOI. Table S3. Significant gray matter thickness differences between HCs and CIA groups, HCs and NOI groups. HCs, healthy controls. CIA, patients with a history of chronic insomnia, whose insomnia symptoms worsened after infection. NOI, individuals with good sleep status had new onset of insomnia after infection.

## Data Availability

No datasets were generated or analysed during the current study.

## Abbreviations

3D-MPRAGE: Three-dimensional magnetization-prepared rapid acquisition gradient echo
BDS: Backward Digit Span
CI: Patients with a history of chronic insomnia whose sleep status did not change after infection
CIA: Patients with a history of chronic insomnia whose insomnia symptoms worsened after infection
COVID-19: Coronavirus disease 2019
DSST: Digit Symbol Substitution Test
DTI-ALPS: Diffusion tensor imaging analysis along the perivascular space
FDR: False discovery rate
FDS: Forward Digit Span
HCs: Healthy controls
ICSD-3: International Classification of Sleep Disorders
ISI: Insomnia Severity Index
LM: Logical Memory
MAGiC: Magnetic resonance image compilation
MRI: Magnetic resonance imaging
NI: Individuals with good sleep quality who had no change in sleep status after infection
NOI: Individuals with good sleep quality who experienced a new onset of insomnia after infection
PD: Proton density
PSQI: Pittsburgh Sleep Quality Index
ROIs: Regions of interest
SARS-CoV-2: Severe acute respiratory syndrome coronavirus 2
SAS: Self-rating Anxiety Scale
SDS: Self-rating Depression Scale
WFT: Word Fluency Test
