# Supplementary material for: Multimodal neuroimaging exploration of the mechanisms of sleep quality deterioration after SARS-CoV-2 Omicron infection
Source: BMC Med. 2024 Jun 26;22:271. doi: 10.1186/s12916-024-03487-9 (PMC11210028; doi:10.1186/s12916-024-03487-9)
Supplement: Supplementary file 1 — Additional file 1. 3D-MPRAGE, MAGiC and DTI scan parameters. [file 12916_2024_3487_MOESM1_ESM.docx]

**Additional file 1:**

Neuroimaging acquisition

Patients were scanned using a 3.0T Premier (GE Signa Architect) and a 48-channel head coil.

The 3D-MPRAGE scanning parameters as follows: 166 sagittal slices, repetition time = 2500 ms, echo time = 3 ms, flip angle = 8°, voxel size = 1 mm × 1 mm × 1 mm, slice thickness = 1 mm, and field of view = 256 mm × 256 mm.

The MAGiC scanning parameters as follows: 36 sagittal slices, repetition time = 4000 ms, echo time = 14.7 ms, voxel size = 1 mm × 1 mm × 4 mm, slice thickness = 4 mm, and field of view = 240 mm × 240 mm.

The DTI were acquired with 3 b valuesand 160 diffusion directions (b=1000, 32 diffusion directions; b=2000, 64 diffusion directions; b=3000, 64 diffusion directions). The analyses of DTI-ALPS were performed with b=1000, and the scanning parameters as follows: 66 sagittal slices, repetition time = 6000 ms, echo time = 1 ms, voxel size = 2 mm × 2 mm × 2 mm, slice thickness = 2 mm, and field of view = 240 mm × 240 mm.

3D-MPRAGE of HCs were acquired using a 3T Siemens Skyra MRI scanner (Siemens Healthcare, Erlangen, Germany) with 32-channel head coil, and the scanning parameters were as follows: 176 sagittal slices, repetition time = 2000 ms, echo time = 2.26 ms, flip angle = 8°, voxel size = 1 mm × 1 mm × 1 mm, slice thickness = 1 mm, and field of view = 256 mm × 256 mm.
