# Supplementary material for: Multimodal neuroimaging exploration of the mechanisms of sleep quality deterioration after SARS-CoV-2 Omicron infection
Source: BMC Med. 2024 Jun 26;22:271. doi: 10.1186/s12916-024-03487-9 (PMC11210028; doi:10.1186/s12916-024-03487-9)
Supplement: Supplementary file 2 — Additional file 2: Table S1. Detailed 46 brain region information. Table S2. Demographic characteristics of groups HCs and CIA, HCs and NOI. Table S3. Significant gray matter thickness differences between HCs and CIA groups, HCs and NOI groups. HCs, healthy controls. CIA, patients with a history of chronic insomnia, whose insomnia symptoms worsened after infection. NOI, individuals with good sleep status had new onset of insomnia after infection. [file 12916_2024_3487_MOESM2_ESM.docx]

**Additional file 2:**

Table S1. Detailed 46 brain region information

| 1 | Precentral_L |
| --- | --- |
| 2 | Precentral_R |
| 3 | Frontal_L |
| 4 | Frontal_R |
| 5 | Rolandic_L |
| 6 | Rolandic_R |
| 7 | Supp_Motor_L |
| 8 | Supp_Motor_R |
| 9 | Olfactory_L |
| 10 | Olfactory_R |
| 11 | Rectus_L |
| 12 | Rectus_R |
| 13 | OFC_L |
| 14 | OFC_R |
| 15 | Insula_L |
| 16 | Insula_R |
| 17 | Cingulate_L |
| 18 | Cingulate_R |
| 19 | ParaHippocampal_L |
| 20 | ParaHippocampal_R |
| 21 | Calcarine_L |
| 22 | Calcarine_R |
| 23 | Cuneus_L |
| 24 | Cuneus_R |
| 25 | Lingual_L |
| 26 | Lingual_R |
| 27 | Occipital_L |
| 28 | Occipital_R |
| 29 | Fusiform_L |
| 30 | Fusiform_R |
| 31 | Postcentral_L |
| 32 | Postcentral_R |
| 33 | Parietal_L |
| 34 | Parietal_R |
| 35 | SupraMarginal_L |
| 36 | SupraMarginal_R |
| 37 | Angular_L |
| 38 | Angular_R |
| 39 | Precuneus_L |
| 40 | Precuneus_R |
| 41 | Paracentral_Lobule_L |
| 42 | Paracentral_Lobule_R |
| 43 | Heschl_L |
| 44 | Heschl_R |
| 45 | Temporal_L |
| 46 | Temporal_R |

Table S2. Summary of participant characteristics.

|  | **HCs(N=15)** | **CIA(N=24)** | **NOI(N=30)** | | | **t/χ^2^/Z** | ***P_1_*** | **t/χ^2^/Z** | ***P_2_*** |
| --- | --- | --- | --- | --- | --- | --- | --- | --- | --- |
| Age (years) | 41.87±13.90^a^ | 40.04±11.75 | 39.80±10.45 | | | 0.44 | 0.663 | 0.508 | 0.616 |
| Gender (male/female)^c^ | 8/7 | 20/4 | 23/7 | | | - | 0.068 | 1.568 | 0.210 |
| Education (years) | 14.93±2.05 | 16.00(12.00, 19.00)^b^ | 15.23±3.01 | | | -0.31 | 0.757 | -0.393 | 0.697 |
| BMI (kg/m^2^) | 23.46±3.30 | 22.05±2.70 | 22.66±4.16 | | | -0.401 | 0.69 | -0.52 | 0.606 |
| Nicotine use (yes/no) | 6/9 | 0/24 | 3/27 | | | - | 0.002 | 3.906 | 0.048 |
| Alcohol use (yes/no) | 4/11 | 3/21 | 3/27 | | | - | 0.396 | 1.036 | 0.309 |
| Handedness | 15R | 24R | 30R | | |  |  |  |  |
| Time interval between Omicron infection and MR examination (days) | - | 81.00±23.42 | 78.83±15.75 | | | - | - | - | - |
| **Vaccination Status** |  |  |  | | |  |  |  |  |
| Unvaccinated | 0 | 1 | 0 | | | 1.357 | 0.904 | 0.111 | 0.946 |
| Single or double vaccinated | 3 | 6 | 5 | | |  |  |  |  |
| Booster vaccinated | 12 | 17 | 25 | | |  |  |  |  |
| **Neuropsychiatric tests** | |  |  |  |  |  |  |  |  |
| PSQI | 5.07±2.19 | 13.12±3.01 | 12.97±2.31 | | | -7.579 | **<0.001^*^** | -9.123 | **<0.001^*^** |
| SAS | 31.27±7.54 | 46.29±10.88 | 47.17±10.49 | | | -4.681 | **<0.001^*^** | -5.223 | **<0.001^*^** |
| SDS | 32.71±7.93 | 48.67±10.83 | 49.20±11.91 | | | -5.128 | **<0.001^*^** | -5.041 | **<0.001^*^** |

*P_1_*: HCs vs CIA. *P_2_*: HCs vs NOI. ^a^ The statistical expression was mean±SD using two-sample t test. ^b^ The statistical expression was M(P_25_, P_75_) using Wilcoxon-Mann‒Whitney U test. ^c^ Chi-square test. HCs, healthy controls. CIA, patients with a history of chronic insomnia whose insomnia symptoms worsened after infection. NOI, individuals with good sleep quality who experienced a new onset of insomnia after infection. N, number of subjects. BMI, body mass index. R, right. PSQI, Pittsburgh Sleep Quality Index. SAS, Self-rating Anxiety Scale. SDS, Self-rating Depression Scale. * *P* values less than 0.05 indicate statistical significance.

Table S3. Significant gray matter thickness differences between HCs and CIA groups, HCs and NOI groups.

| **Index** | **Brain regions** | **Side**  **(R/L)** | **Size** | **MNI coordinate** | | | **Values** | | ***P*** |
| --- | --- | --- | --- | --- | --- | --- | --- | --- | --- |
|  |  |  |  | **X** | **Y** | **Z** |  |  |  |
| **HCs vs CIA (15 vs 24)** | | | | | | | |  |  |
| Thickness(mm) | inferior parietal | L | 3463.64 | -39.0 | -61.2 | 17.2 | 2.29±0.09 | 2.53±0.08 | <0.001 |
|  | lingual | L | 846.92 | -6.0 | -68.4 | 0.7 | 1.86±0.08 | 1.64±0.13 | <0.001 |
|  | lateral orbitofrontal | L | 501.86 | -13.1 | 24.5 | -16.4 | 2.40±0.11 | 2.21±0.11 | <0.001 |
|  | superior parietal | L | 475.44 | -17.4 | -76.0 | 34.5 | 1.95±0.09 | 2.18±0.17 | <0.001 |
|  |  |  | 252.67 | -16.1 | -65.6 | 47.8 | 2.07±0.14 | 2.38±0.19 | <0.001 |
|  | pericalcarine | R | 1322.07 | 6.1 | -75.5 | 4.5 | 1.81±0.08 | 1.62±0.08 | <0.001 |
|  | superior parietal | R | 600.02 | 16.1 | -70.1 | 51.9 | 2.14±0.10 | 2.41±0.13 | <0.001 |
| **HCs vs NOI (15 vs 30)** | | | | | | | |  |  |
| Thickness(mm) | lingual | L | 822.75 | -5.7 | -82.9 | 1.0 | 1.91±0.10 | 1.68±0.12 | <0.001 |
|  | lateral orbitofrontal | L | 757.01 | -19.6 | 31.3 | -14.4 | 2.51±0.18 | 2.27±0.13 | <0.001 |
|  | superior parietal | L | 690.70 | -17.5 | -75.2 | 34.0 | 2.07±0.10 | 2.29±0.13 | <0.001 |
|  |  |  | 442.41 | -21.2 | -64.9 | 52.6 | 2.07±0.15 | 2.35±0.12 | 0.001 |
|  | inferior parietal | L | 589.56 | -29.2 | -82.1 | 12.3 | 2.07±0.13 | 2.27±0.09 | <0.001 |
|  |  |  | 560.42 | -39.4 | -61.1 | 15.9 | 2.25±0.17 | 2.47±0.11 | <0.001 |
|  | lateral occipital | L | 373.09 | -21.2 | -64.9 | 52.6 | 2.07±0.14 | 2.28±0.13 | <0.001 |
|  | cuneus | L | 331.75 | -3.4 | -91.7 | 13.4 | 2.01±0.19 | 1.76±0.20 | <0.001 |
|  | pericalcarine | R | 2807.91 | 18.4 | -70.8 | 10.7 | 1.86±0.07 | 1.66±0.10 | <0.001 |
|  | superior parietal | R | 671.45 | 18.4 | -67.3 | 50.2 | 2.09±0.11 | 2.33±0.10 | <0.001 |

R: right. L: left. MNI: Montreal Neurological Institute. HCs, healthy controls. CIA, patients with a history of chronic insomnia, whose insomnia symptoms worsened after infection. NOI, individuals with good sleep status had new onset of insomnia after infection.
